# Patient-Specific Virtual Surgical Planning and In-House CAD-/CAM-Guided Vascularized Bone Flaps for Salvage Extremity Reconstruction: A Case Series

**DOI:** 10.3390/bioengineering13070721

**Published:** 2026-06-24

**Authors:** Jaideep Seth, Matthew D. Marquardt, Rachel Herster, Teri Snyder, David W. Nash, John Alexander, Angela C. Collins, Jason M. Souza, Humza S. Shaikh, Juan E. Santiago-Torres, Laura S. Phieffer, Tobin Eckel, Kyle VanKoevering

**Affiliations:** 1 College of Medicine, The Ohio State University, Columbus, OH 43210, USA; 2 Center for Design and Manufacturing Excellence, The Ohio State University, Columbus, OH 43210, USA; 3 Department of Plastic and Reconstructive Surgery, The Ohio State University Wexner Medical Center, Columbus, OH 43210, USA; 4 Department of Orthopaedics, The Ohio State University Wexner Medical Center, Columbus, OH 43210, USA; 5 Department of Otolaryngology—Head and Neck Surgery, The Ohio State University Wexner Medical Center, Columbus, OH 43210, USA

**Keywords:** vascularized bone flap, virtual surgical planning (VSP), 3D-printing, bone defect reconstruction, bone nonunion

## Abstract

The surgical management of extremity bone defects, particularly post-traumatic nonunion wounds, remains a challenge. Vascularized bone flaps (VBFs), widely used for mandibular reconstruction in head and neck oncologic surgery, are less established in extremity reconstruction and are typically performed freehand, which has several limitations. In the past decade, virtual surgical planning (VSP) and computer-aided design and modeling (CAD-CAM) technology have enabled patient-specific 3D-printed models to guide reconstruction. While this technology has been used extensively in head and neck reconstructive surgery, its application to extremity reconstruction is less well-documented. This case series evaluates the feasibility, safety, and surgical utility of VSP and in-house CAD-CAM manufacture of 3D-printed models and cutting guides for post-traumatic non-healing extremity reconstructions using VBFs. Eight patients at a single tertiary academic center underwent VBF reconstruction guided by patient-specific models and cutting guides, with cases grouped into categories (humerus, femur, and tibia). The multi-disciplinary workflow supported preoperative visualization, osteotomy planning, and intraoperative execution. All vascularized flaps survived, and radiographic union was documented in patients with adequate follow-up. These findings suggest that integrating VSP and CAD-CAM into trauma-associated VBF extremity reconstruction is feasible and safe and may improve reconstructive accuracy and enhance multi-disciplinary team workflow, potentially contributing to improved clinical outcomes.

## 1. Introduction

The surgical management of delayed and nonhealing fractures due to traumatic large, long-bone segmental defects is challenging, often requiring multiple surgeries and innovative solutions to restore form and function. Traditionally, large segmental defects have been reconstructed with allogeneic bone grafts, the Masquelet technique, or autologous bone grafting with instruments such as the Reamer–Irrigator–Aspirator system [1,2,3,4]. Large segmental reconstructions are typically performed using freehand techniques where the surgeon manually shapes and positions a bone graft and internal fixation based on intra-operative fluoroscopy and direct visualization. This technique has several limitations, including a relatively high risk for nonunion, allograft fracture, and infection [5,6]. Studies have shown that large segmental allografts are associated with complications in 76% of patients and a graft-related failure rate of 15% [7].

Vascularized bone flaps (VBF’s) utilizing autogenous fibular bone represent an under-utilized approach to orthopedic reconstruction with the potential to improve union rates by preserving the blood supply to the graft through microvascular anastomoses, and it has the ability to address composite soft tissue defects if necessary [8,9]. This approach has been shown to be effective in treating nonunions, especially those with significant osseous defects [10]. However, most vascularized extremity reconstruction procedures still rely on a “freehand” contouring and sizing technique, without the assistance of patient-specific guides or models to optimize fit. VBF’s require highly precise pedicle orientation and stable fixation to maximize outcomes, further complicating an already challenging procedure.

In the past decade, virtual surgical planning (VSP) combined with computer-aided design and computer-aided modeling (CAD-CAM) technology has enabled the low-cost and convenient production of patient-specific 3D-printed surgical models [11]. These models are widely used in head and neck reconstructive surgery, where they assist the surgical team during VBF reconstruction of complex head and neck bone defects as a result of tumor resection, infection, osteoradionecrosis, or trauma [12,13,14,15,16]. Utilizing CAD-CAM technology, surgeons are able to preoperatively and intraoperatively visualize complex anatomical defects, digitally execute a planned surgical procedure in CAD to simulate surgical interventions, and create patient-specific anatomical models and cutting guides to execute the surgical procedure as planned [17]. Patient-specific models can provide the surgical team with an exact “native” model—i.e., the bony structure demonstrating the tumor or defect—or the “reconstructed” model—i.e., the planned result after intended reconstruction—as tangible representations of the patient’s anatomy. Cutting guides are customized according to patient-specific templates to ensure precise, reproducible osteotomies of native and allograft or autogenous bone.

While the use of VSP and CAD-CAM technologies has been widely adopted for osseous flaps in maxillofacial and craniofacial surgeries, their application to extremity reconstruction using vascularized bone flaps remains comparatively limited. Prior studies of extremity defects using 3D-printed patient-specific cutting guides have shown improved accuracy in femoral osteotomies [18,19]. Additionally, the head and neck reconstruction literature has shown that using VSP to produce custom cutting guides and splint-guided surgery for mandibular reconstruction was associated with improved surgical efficiency, as measured by reduced operating and ischemic time, and higher rates of bony union [20,21,22].

Building on these established benefits, this case series explores eight cases where VSP and CAD-CAM manufacturing of 3D-printed patient-specific surgical models and cutting guides were used for vascularized bone flap reconstruction of traumatic, non-healing extremity bone defects. To our knowledge, this is the first case series examining the utilization of VSP and CAD-CAM technology in extremity defects in trauma salvage cases involving VBFs. By detailing the engineering and planning processes and clinical outcomes, this case series aims to demonstrate the safety of this technology and its potential to improve surgical efficiency and achieve accurate bony alignment.

## 2. Materials and Methods

### 2.1. Patient Selection and Study Design

A retrospective chart review was undertaken on eight patients from 2022 to 2025 who underwent VBFs for limb-salvage reconstructions at a single academic, tertiary care institution with the aid of in-house CAD-/CAM-produced patient-specific anatomic models and cutting guides. The population included patients who had developed nonunion or delayed fracture healing from prior failed reconstruction of traumatic injuries. Each patient underwent reconstruction with VBFs using either a single or double-barreled free fibula flap.

### 2.2. In-House CAD-CAM Production

**Figure 1:**
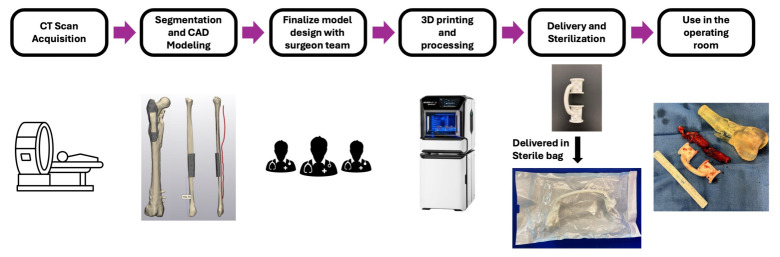
Overview of the 3D-printed anatomic model and cutting guide production process.

Our institution operates a well-established in-house service for producing patient-specific anatomic models and guides. A dedicated quality management system ensures quality control throughout the design and production process. The process begins with the acquisition of a pre-operative computed tomography (CT) scan [23]. Uncompressed Digital Imaging and Communications in Medicine (DICOM) data are then imported into FDA 510(K)-cleared segmentation and 3D modeling software [MIMICS Innovation Suite, Materialise, Leuven, Belgium], which is specifically indicated for generating three-dimensional models from medical imaging data. Using this software, the affected bone and its defect from each scan are segmented through a thresholding technique, smoothed, and converted into a stereolithography (STL) file for both the injury site and the intended donor site. Cutting guides are produced using Materialise 3-matic (version 18.0). After the patient-specific model is segmented from the CT scan, a 3 mm global offset is applied for fibula guides (to account for the muscle cuff of the donor bone), but no offset is applied to the primary surgical site, as dissection is performed in a subperiosteal plane. The virtual surgical plan is created with both orthopedic and plastic surgical input. Each plane is highly individualized to each patient, balancing the size of the donor bone, the extent of devitalized bone that needs to be removed, and the preservation of key anatomy such as an articular surface. The resulting surgeon-specified osteotomy planes are positioned to cut the bone model, and curve-based surfaces are created that conform precisely to the osseous surface anatomy. These surfaces are extruded externally to create bases that extend proximal or distal to the osteotomy plane. Flanges at the osteotomy plane provide additional stability or capture for the oscillating saw, guide bridges connect the two bases, and guide tubes allow for Kirshner wires to be placed to hold the guide to prevent motion during the osteotomy. Native anatomical models were also provided for every case to allow for adjustments as needed. The overall production process is outlined in Figure 1.

The resulting virtual models of the bones and the cutting guides were then 3D-printed using commercially available stereolithography (SLA) [Form 3B, Formlabs, Somerville, MA, USA] and material jetting (J5 MediJet, Stratasys, Eden Prairie, MN, USA) technologies. BioMed Amber resin was utilized for the single-color SLA 3D-printed models, and BioMed White resin was used for the cutting guides. VeroCyanV, VeroMagentaV, VeroYellowV, DraftWhite, and MED610 Biocompatible Clear (Stratasys, Eden Prairie, MN, USA) were used for multi-colored or larger-sized material jetting 3D prints. Both sets of print materials have undergone biocompatibility testing. The anatomic models and guides were subsequently sterilized for intraoperative use via autoclave.

### 2.3. Outcome Assessment

Patient charts were reviewed to gather pertinent pre-operative history, 3D tools produced, surgical approach, and post-operative outcomes and complications. This retrospective study was approved by the institutional review board (2023C0216). The requirement for informed consent was waived due to the retrospective design and use of de-identified data. Surgical characteristics included reconstruction plan, anatomic defect location and donor site, vascular anastomoses, and ischemia time. Postoperative outcomes included both perioperative complications, such as infection and flap survival, and long-term outcomes, including union rates and functional outcomes. Post-operative X-ray frequency to monitor bony union was determined by the surgeon depending on the extremity involved and patient history.

## 3. Results

**Figure 2:**
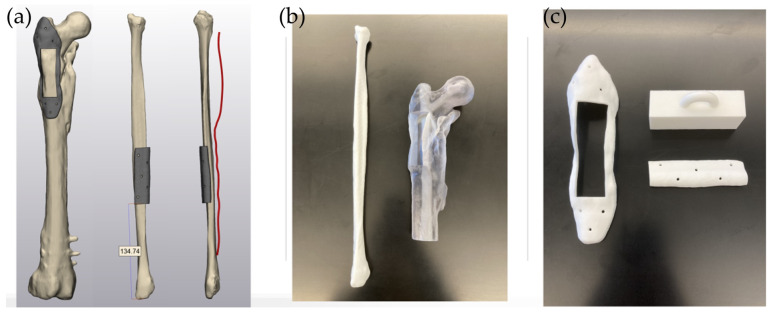
From left to right: (**a**) virtual CAD cutting guide models placed on patient-specific anatomy, (**b**) 3D-printed patient-specific anatomic models, and (**c**) 3D-printed patient-specific cutting guides.

**Figure 3:**
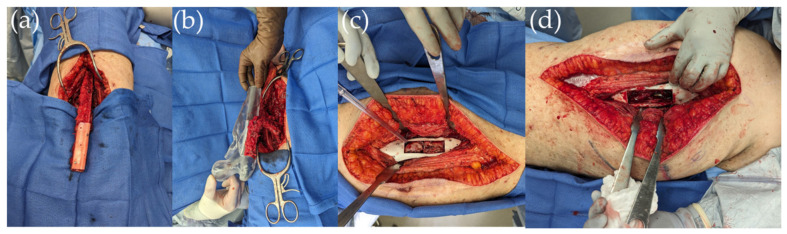
Series of images demonstrating how 3D-printed anatomic models and cutting guides were used for a femur reconstruction. From left to right: (**a**) cutting guide placed on the graft femur, (**b**) the fibula graft alongside the patient-specific femur model, (**c**) the femur cutting guide prior to bone removal, and (**d**) femur guide after bone removal prior to addition of the graft.

In total, eight cases were included in this case series, with pertinent background information, surgical characteristics, and outcome data summarized in Table 1, along with one illustrative case involving the tibia, femur, and humerus explored in greater detail in Supplementary Files Case S1, Case S2, and Case S3, respectively. Figure 2 and Figure 3 demonstrate how a 3D-printed anatomic model and cutting guide were manufactured and used for one of the cases.

Across the eight cases, seven were trauma-related defects, and one was a nonunion due to a radiation-associated pathologic fracture. All patients had failed prior surgical management and presented with severe functional impairments, including inability to ambulate or soft-tissue compromise. All cases involved a single- or double-barrel vascularized fibula osseous alone flap. All cases utilized one or more patient-specific 3D-printed models or cutting guides. Ischemia time ranged from 108 to 209 min. Only one patient had a post-operative complication related to the procedure. This patient developed a hematoma beneath a previously placed anterolateral thigh free flap, which was drained on postoperative day six without further sequelae. Two patients had complications that were likely unrelated to the procedure. One patient who underwent a fibula osseous alone free flap had a complication nine months postoperatively when they developed pyogenic arthritis and an edematous ankle. Another patient died of sepsis related to mitral and tricuspid endocarditis at six months postop, likely due to the chronic infection that they presented with and their IV drug use history. The vascularized fibula flap survived in all eight cases. Documented radiographic union was achieved in two patients, with a third at near-complete union, and progressive healing was documented in the remaining patients who presented with adequate follow-up. Union could not be assessed in the patient who died or who was lost to follow-up. Importantly, according to patient-reported outcomes obtained at follow-up visits, no patients experienced extremity function deterioration attributable to the reconstruction.

## 4. Discussion

Vascularized bone reconstruction offers distinct advantages over allogenic bone grafts, particularly in the management of fracture nonunion and compromised healing environments. Vascularized grafts provide an immediate, viable blood supply, which enhances graft viability, accelerates union, and improves resistance to infection—key factors in cases of poor local vascularity, extensive soft tissue loss, or active infection [24,25,26]. Meta-analyses and systematic reviews demonstrate that vascularized fibular grafts, either alone or combined with allograft, result in lower nonunion rates and improved healing compared to allograft alone, with a nonunion rate of 13% versus 21.4% for allograft alone in oncologic lower extremity reconstruction [27,28]. Taken together, the data suggests that vascularized bone reconstruction would have significant value in treating nonhealing wounds, especially for limb salvage in the setting of large segmental defects. Notably, every patient in this case series had failed prior standard-of-care reconstruction and presented with a complex segmental defect rather than a simple diaphyseal gap.

Virtual surgical planning (VSP) and the integration of computer-aided design and manufacturing (CAD/CAM) technologies have significantly advanced the precision and efficiency of complex skeletal reconstructions, specifically in the realm of oral and maxillofacial surgeries [11,12,13]. In this case series, we present orthopedic trauma salvage cases in which surgeons elected to use VSP due to complex and large osseous defects that necessitated vascularized bone flaps. We provide initial evidence for the safety, feasibility, and surgical utility of VSP with 3D-printed surgical guides/models for these extremity bone defect reconstructions, leveraging vascularized bone flaps. The outcomes of these cases suggest the potential of this approach in upper and lower extremity bone defects in assisting both the surgical team and workflow.

The use of VSP and 3D-printed models to improve geometric accuracy has been detailed across multiple bony defect locations. In fibula free flap reconstruction of the mandible, patient-specific cutting guides were associated with reduced angular and distance deviations compared to free-hand techniques [29]. Improved cutting accuracy due to patient-specific cutting guides has also been shown in orthopedic osteotomies [30]. Our case series is consistent with this literature, supporting the potential for improved accuracy in the reconstruction of extremity bony defects. As shown in prior literature, geometric accuracy is essential for achieving osseous union [22].

All surgeons involved in this case series reported the impression that the use of VSP and 3D modeling for reconstruction of large segmental defects reduced ischemia time relative to cases without the benefit of this technology. However, this observation is reported as hypothesis-generating for future studies rather than as a conclusion or evidence of benefit. No studies in the orthopedic space have explored this question, but studies on the reconstruction of osseous defects in head and neck and craniofacial surgery have shown that VSP and 3D-printed guides can streamline workflows and minimize intraoperative trial-and-error graft contouring. Specifically, one study showed a mean reduction in ischemia time by 36 min with the use of VSP and 3D-printed guides [31]. Through illustrative case 2 (Supplement S2), we describe the intraoperative workflow in detail. Ischemia times were recorded for all cases and ranged from 108 to 209 min. In the absence of a comparison group, we cannot interpret these as reduced relative to conventional approaches. To our knowledge, no large meta-analysis has been done to examine the relationship between ischemia time and VSP-/3D-printed surgical guides. All flaps survived, and continuing bony healing or union was documented in patients who reached adequate follow-up. While these complex limb-salvage cases are often very challenging, this preliminary data suggests that the combination of custom cutting guides and vascularized bone reconstructions to enhance reconstructive accuracy may improve the probability of successful limb salvage. We do not interpret these outcomes as evidence of the superiority of VSP over conventional methods; however, VSP contributed to these outcomes by facilitating 3D preoperative planning and case-specific osteotomy planning in anatomical regions with complex defects.

This case series also demonstrates how VSP and 3D-printed surgical modeling can facilitate preoperative visualization and osteotomy planning across multi-specialty surgical teams, including both orthopedic and plastic surgeons, in a truly collaborative “orthoplastics” care team model. All illustrative cases (Supplement S1–S3) highlight how this technology integrates into the workflow of orthoplastics teams. Prior studies in head and neck reconstructive surgery have shown that in-house, surgeon-directed VSP and 3D-printing enabled multidisciplinary teams to rapidly iterate and customize surgical guides and implants, improving surgical planning and efficiency and reducing costs [32]. This case series suggests that such advantages might also translate to orthoplastics teams performing complex limb-salvage surgery.

Patient safety is another important variable explored in this case series. Across all cases, there were no incidences of infection or adverse events attributed to the models or guides. This aligns with prior reports in the craniofacial and orthopedic literature, where sterilizable, biocompatible 3D-printed models have shown similar safety profiles [33,34]. Additionally, no nonunion was observed in patients with adequate follow-up, suggesting that patient-specific 3D-printed tools have the potential to augment long-term success in extremity reconstruction cases.

This study has several limitations, with the most notable being the small sample size. Additionally, this case series is only descriptive. A comparative analysis between limb-salvage cases using VBFs with or without the use of VSP and CAD-CAM technology should be undertaken to determine the true benefit of 3D-printed patient-specific cutting guides and models. In this study, neither the number of adjustments, adjustment time, nor ischemia time was standardized. While surgeons reported needing fewer adjustments and felt that adjustment and ischemia times were comparatively lower, this was not directly or objectively measured. It is possible that the precise preoperative measurements enabled by VSP could expedite complex reconstructions and reduce flap ischemia time; however, future trials should evaluate these endpoints.

## 5. Conclusions

Based on our knowledge, this is the first case series report to examine the utilization of “in-house” CAD-/CAM-produced patient-specific cutting guides for vascularized bone flaps of large extremity defects in the setting of traumatic nonunion salvage operations. These cases demonstrate that CAD-/CAM-produced patient-specific models and cutting guides might offer a promising and efficient approach to addressing large segmental osseous defects using vascularized bone flaps. The outcomes reported herein suggest that this technique is safe and might improve reconstructive accuracy and efficiency. It also establishes that VSP and patient-specific 3D-printed cutting guides can be feasibly integrated into vascularized bone flap reconstruction of complex extremity defects while providing insights into workflow and endpoints for future controlled studies comparing against traditional approaches.

## Figures and Tables

**Table 1:** Overview of demographics, surgical characteristics, and clinical outcomes for each patient.

Site	Demographic Information	Injury	Clinical Presentation	Initial Management That Failed	Reconstruction Plan	3D Tools Created	Anastomotic Anatomy	Ischemia Time	Post Op Complications	Flap Survival	Healing Result	Long-Term Functional Outcome	Illustrative Case #
Tibia	29 year old male	Type 3 open left tibia-fibula fracture secondary to a motor vehicle collision two years prior	Has slight pain, but able to ambulate with a limp	Irrigation, debridement, and open reduction with internal fixation with a post-op course complicated by infection, delayed wound healing, and eventual hardware explantation	Stage 1: Right thigh free ALT flap with placement of antibiotic spacer Stage 2 (a few months later): Right vascularized free fibula flap	Left tibia anatomical model Right fibula anatomical model Tibia outer cutting guide Tibia inner cutting guide Fibula guide	Right peroneal artery to the left posterior tibial artery Peroneal VC to the vena comitantes of the posterior tibia	108 min	Hematoma underneath ALT free flap drained of post-op day 6	Yes	Near-complete bony union	Ambulating independently without assistive devices	1
Tibia	38 year old female	Left tibial nonunion secondary to left proximal tibial fracture complicated by infection	Lower left extremity pain and erythema without numbness or tingling	Initial tibial fracture complicated by infection requiring multiple incision and drainage procedures with skin grafting. An external fixation with antibiotic spacer was then complicated by infected non-union. Was then managed by circular frame which was complicated by non-compliance and incarceration.	Stage 1: Left tibia incision and drainage with placement of antibiotic spacer Stage 2 (a few months later): Vascularized double barrel fibula	Right Fibula	Peroneal artery to the posterior tibial artery and the saphenous vein to the vena comitans of the posterior tibial artery.	120 min	Infection seen 4 months post-op at site of pins but infectious workup was unrevealing	Yes	Continued signs of healing but fracture gap still evident 4 months post surgery.	Passed away at 6 months post-surgery due to multi-organ failure in the setting of sepsis and mitral and tricuspid valve endocarditis. At 4 months was able to ambulate with weight bearing as tolerated.	N/a
Tibia	54 year old male	Right distal tibia nonunion secondary to motor vehicle collision complicated by infection	Chronic pain to the right ankle with chronic sinus draining of the right knee. Limited mobility and limited flexion of the knee.	Fracture initial fixed with external fixation and intramedullary nails with soleus muscle flap. This was complicated by an ankle infection that was treated with I&D and a skin flap. Then complicated with knee and ankle infection and treated with I&D and placement of IMN with an antibiotic spacer placed.	Additional debridement, hardware removal and a TTC nail with fusion and free fibula flap	Right tibia + talus (native anatomy) Right tibia + talus (defect removed) Left fibula Tibia cutting guide Fibula guides (proximal and distal segments for double barrel recon)	Peroneal artery to the posterior tibial artery and the peroneal vein to the vena comitans of the posterior tibial artery.	142 min	Yes; pyogenic arthritis and swollen ankle 6/13/25	Yes	Patient was lost to follow up from orthoplastics team after 4 week visit	Patient was lost to follow up from orthoplastics team after 4 week visit	N/a
Femur	59 year old female	Chronic right femoral nonunion secondary to right substrochanteric femur fracture two years prior complicated by infection	Pain with ambulation and able to walk with assistance from a walker	ORIF with intertrochanteric intramedullary rod that became infected and was further complicated by delayed wound healing	Right Vascularized Fibula Flap	Right femur anatomical model with bone gap Right fibula anatomical model Femur outer cutting guide Femur inner cutting guide Fibula guide	Peroneal artery to the descending branch of lateral circumflex femoral artery Vena comitans of the peroneal system to the descending branch of lateral circumflex femoral system	8 min for flap insert time with total ischemia time of 119 min)	None	Yes	Radiographic union at 13 months	Ambulating with a rollator assistive device	2
Femur	36 year old male	Right distal femur fracture secondary to motor vehicle accident with signs of bone loss complicated by infection	Unable to ambulate on the right lower extremity due to pain	Femur resection with antibiotic beads and bone grafting from the iliac crest and lateral plate in Africa.	Debridement procedure first followed by vascularized bone reconstruction with ipsilateral fibula	Right Distal Femur Right Fibula	Superficial femoral artery to the peroneal artery and the superficial femoral vein to the saphenous vein.	165 min	None	Yes	Fracture appeared stable with increasing consolidation posteriorly and laterally	Able to ambulate with crutches without pain or difficulty	N/a
Femur	61 year old female	Right distal femur nonunion secondary to fracture complicated by infection	Pain with ambulation and deformity of right leg	Patient had undergone two unsuccessful intramedullary nail procedures without bony union	Left vascularized fibula flap	Dital Right Femur Left Fibula Guide	Peroneal artery to the deep femoral circumflex artery and peroneal vein to the vena comtans of the deep femoral circumflex artery.	118 min	None	Yes	Graft healing but slowly with evidence of new bone formation	Noted feelings of instability in knee but no pain	N/a
Femur	58 year old female	Left femoral nonunion secondary to radiation-associated pathologic fracture with evidence of hardware failure	Able to ambulate with a walker and bear weight but pain is present and stable	Open biopsy and cephalomedullary nail fixation	Vascularized free fibula autograft with removal of hardware and revision open reduction internal fixation	Left Femur Left Fibula	Peroneal artery to the side branch of the superficial femoral artery and peroneal vein to the side branch of the superficial femoral vein	130 min	None	Yes	Progressive healing of the fracture site	Uses a wheelchair. Ambulate sonly short distances due to back pain not related to surgery.	N/a
Humerus	17 year old male	Segmental bone loss of the distal humerus and significant soft tissue injury due to an ATV accident	Slight pain, but arm in a cast on presentation	Initial reconstruction with bone transport was complicated by fungal infection	Double barrel vascularized fibula	Left humerus anatomic model Left fibula anatomic model Left fibula cutting guide	(1) Venous loop: Descending branch of the lateral circumflex femoral artery to the left brachial artery PLUS the lateral circumflex femoral vein to the brachial vein (2) Peroneal artery to the descending branch of the lateral circumflex femoral arterial graft	209 min	None	Yes	Radiographic union at 11 months	No pain at rest but slight pain that is activity-driven. Improved wrist strength and range of motion	3

## Data Availability

The original contributions presented in this study are included in the article/Supplementary Material. Further inquiries can be directed to the corresponding author.

## Supplementary Materials

The following supporting information can be downloaded at: https://www.mdpi.com/article/10.3390/bioengineering13070721/s1, Case S1: Illustrative Tibia Reconstruction; Case S2: Illustrative Femoral Nonunion Reconstruction; Case S3: Illustrative Humerus Reconstruction.
