# Supplementary material for: Patient-Specific Virtual Surgical Planning and In-House CAD-/CAM-Guided Vascularized Bone Flaps for Salvage Extremity Reconstruction: A Case Series
Source: Bioengineering (Basel). 2026 Jun 24;13(7):721. doi: 10.3390/bioengineering13070721 (PMC13405651; doi:10.3390/bioengineering13070721)
Supplement: Supplementary file 1 [file bioengineering-13-00721-s001.zip › bioengineering-4323522-supplementary.pdf]

Supplementary Material S1

Case S1: Illustrative Tibia Reconstruction

Case 1 demonstrates the application of patient-specific 3D modeling and osteotomy guides in a complex tibia reconstruction using a vascularized free fibula flap. The patient was a 29 year old male with history of a type 3 open left tibia-fibula fracture secondary to a motor vehicle collision two years prior. Initial management included irrigation, debridement, and open reduction with internal fixation, but the postoperative course was complicated by infection, delayed wound healing, and eventual hardware explantation.

Following discussion at our institution's multidisciplinary orthoplastic reconstruction conference, a two-stage approach was planned. The first stage involved a free ALT flap from the right thigh and placement of an antibiotic spacer. The second stage occurred a few months later and comprised a contralateral vascularized free fibula flap for definitive osseous reconstruction utilizing patient-specific cutting guides. Preoperative preparation included meeting with the engineers at our in-house 3D printing lab to design the patient-specific 3D models and cutting guides. Five components were printed: left tibia anatomical model, right fibula anatomical model, tibia outer cutting guide, tibia inner cutting guide, and fibula guide. The anatomic models enabled detailed preoperative visualization of the defect and surrounding native bony architectures, while also serving as anatomically accurate substrates to verify cutting guide placement and fit.

Intraoperatively, the tibial defect was prepared using the custom guides to ensure precise osteotomies and optimal alignment. Traditionally, freehand graft contouring would have required repeated intraoperative measurements and adjustments until the graft and defect fit. In contrast, the fibula cutting guide permitted a single, accurate cut and first-pass inset, eliminating iterative shaping and minimizing handling of the vascular pedicle. The right peroneal artery and the peroneal VC which were then anastomosed under a microscope with the left posterior tibial artery and the vena comitantes of the posterior tibia, respectively.

At six month follow-up, the patient demonstrated radiographic evidence of near-complete bony union and was ambulating independently without assistive devices. Demographic and procedural details are summarized in Table 1. Overall, this case highlights how patient-specific 3D models and cutting guides facilitated graft geometric accuracy during defect reconstruction. Successfully achieving a precise fit is a key outcome for reducing the risk of malalignment-related nonunion.

Supplementary Material S2

Case S2: Illustrative Femoral Nonunion Reconstruction

Case 2 demonstrates the application of patient-specific 3D modeling and osteotomy guides in a complex femur reconstruction using a vascularized free fibula flap. A 59-year-old female presented with a chronic right femoral nonunion that had persisted for more than two years, resulting in significant functional limitation and inability to ambulate.

Initial management included ORIF with intertrochanteric intramedullary rod that became infected and was complicated by delayed wound healing. Prior to our reconstruction with an ipsilateral vascularized fibula bone flap, every care was taken to ensure removal of residual infection. Preoperative preparation included collaboration between the engineers at our in-house 3D printing lab with the reconstructive team. Five components were 3D printed: Right femur anatomical model with bone gap, right fibula anatomical model, Femur outer cutting guide, Femur inner cutting guide, and fibula guide. The left femur and right fibula 3D anatomic models provided a detailed spatial understanding of the bony defect and permitted preoperative verification of guide positioning and contour.

Intraoperatively, one surgeon resected the callus and prepared the defect using the custom femur cutting guides, while another surgeon harvested the fibular flap. Fibular osteotomies were made approximately 8 cm proximal and distal to the fibular tip and head respectively and the fibular flap was cut by placing the cutting guide on the distal end of the fibula. Time from division of the vessels to definitive inset of the fibular strut was 8 minutes, which is significantly shorter than is typical due to the elimination of repeated intraoperative measurements and adjustments prior to achieving optimal fit. The peroneal artery and the vena comitans of the peroneal system were preserved on the bone flap and anastomosed under microscope to the descending branch of lateral circumflex femoral artery and the descending branch of lateral circumflex femoral system, respectively.

Post operatively, the patient was ambulating with a rollator assistive device by 6 months and radiographic union was observed by 13 months. Demographic and procedural details are summarized in Table 1. This case illustrates how patient-specific 3D models and cutting guides have the potential to reduce ischemia time by minimizing intraoperative trial-and-error, even during complex femoral reconstructions.

Supplementary Material S3

Case S3: Illustrative Humerus Reconstruction

Case 3 illustrates how patient-specific 3D modeling and osteotomy guides can be utilized for complex humerus reconstruction using a vascularized free fibula flap in a double barrel configuration. A 17 year old male sustained segmental bone loss of the distal humerus and significant soft tissue injury due to an ATV accident. Initial reconstruction with bone transport was complicated by fungal infection, necessitating abortion of the procedure. Definitive surgery included a vascularized bone flap via an AV loop from the proximal brachial artery due to prior injury to his brachial artery.

Preoperative collaboration between the reconstructive team and our 3D printing lab produced patient-specific 3D models of the Left humerus, left fibula, left fibula guide. The anatomic models of the left humerus and left fibula facilitated preoperative visualization of the defect and enabled simulation of the osteotomies to ensure proper placement and fit.

Intraoperatively, standard approaches for humerus and fibula reconstruction were followed. When the brachial artery was determined to be a suitable vascular recipient, the descending branch of the lateral circumflex femoral artery and the lateral circumflex femoral vein from the anterior lateral thigh were harvested. The descending branch of the lateral circumflex femoral artery was anastomosed to the left brachial artery in end-to-side fashion while the lateral circumflex femoral vein was anastomosed to the brachial vein. The two vessels were then anastomosed with a venous coupler to create a venous loop. The distal humerus site was prepared by removing the antibiotic spacer and freshening the osteotomies both proximally and distally. When harvesting the fibula, approximately 6 cm of fibula were preserved proximally and distally with the aid of a custom cutting guide and then placed into the humerus inset in a double-barreled fashion and fixated with struts. Finally, the peroneal artery was anastomosed in an end-to-end fashion to the descending branch of the lateral circumflex femoral arterial graft.

Post-operative course was notable for restoration of function and radiographic boney union within 11 months. However, fusion of the ulnohumeral joint necessitated a synostosis takedown surgery at 13 months post-op. Demographic and procedural details are summarized in Table 1. This case illustrates the adaptability of patient-specific cutting guides to anatomically complex regions in the upper extremity and underscores the versatility of the vascularized fibular free flap as a reconstructive option even in the setting of compromised recipient vasculature.
